# Supplementary material for: A Critical View of the Application of the APEX Software (Aqueous Photochemistry of Environmentally-Occurring Xenobiotics) to Predict Photoreaction Kinetics in Surface Freshwaters
Source: Molecules. 2019 Dec 18;25(1):9. doi: 10.3390/molecules25010009 (PMC7017383; doi:10.3390/molecules25010009)
Supplement: Supplementary file 1 [file molecules-25-00009-s001.zip › 3.2.4_gcc-4.4.0.zip!/3.2.4_gcc-4.4.0/3.2.4_gcc-4.4.0/doc/octave/html/interpreter/Function-Headers.html]

Function Headers - Untitled


Next: Documentation Tips,
Previous: Comment Tips,
Up: Tips and Standards


---

### C.4 Conventional Headers for Octave Functions

Octave has conventions for using special comments in function files
to give information such as who wrote them. This section explains these
conventions.

The top of the file should contain a copyright notice, followed by a
block of comments that can be used as the help text for the function.
Here is an example:

```
     ## Copyright (C) 1996, 1997, 2007 John W. Eaton
     ##
     ## This file is part of Octave.
     ##
     ## Octave is free software; you can redistribute it and/or
     ## modify it under the terms of the GNU General Public
     ## License as published by the Free Software Foundation;
     ## either version 3 of the License, or (at your option) any
     ## later version.
     ##
     ## Octave is distributed in the hope that it will be useful,
     ## but WITHOUT ANY WARRANTY; without even the implied
     ## warranty of MERCHANTABILITY or FITNESS FOR A PARTICULAR
     ## PURPOSE.  See the GNU General Public License for more
     ## details.
     ##
     ## You should have received a copy of the GNU General Public
     ## License along with Octave; see the file COPYING.  If not,
     ## see <http://www.gnu.org/licenses/>.
     
     ## usage: [IN, OUT, PID] = popen2 (COMMAND, ARGS)
     ##
     ## Start a subprocess with two-way communication.  COMMAND
     ## specifies the name of the command to start.  ARGS is an
     ## array of strings containing options for COMMAND.  IN and
     ## OUT are the file ids of the input and streams for the
     ## subprocess, and PID is the process id of the subprocess,
     ## or -1 if COMMAND could not be executed.
     ##
     ## Example:
     ##
     ##  [in, out, pid] = popen2 ("sort", "-nr");
     ##  fputs (in, "these\nare\nsome\nstrings\n");
     ##  fclose (in);
     ##  while (ischar (s = fgets (out)))
     ##    fputs (stdout, s);
     ##  endwhile
     ##  fclose (out);
```

Octave uses the first block of comments in a function file that do not
appear to be a copyright notice as the help text for the file. For
Octave to recognize the first comment block as a copyright notice, it
must start with the word `Copyright' after stripping the leading
comment characters.

After the copyright notice and help text come several header
comment lines, each beginning with ## header-name:. For
example,

```
     ## Author: jwe
     ## Keywords: subprocesses input-output
     ## Maintainer: jwe
```

Here is a table of the conventional possibilities for header-name:

Author: This line states the name and net address of at least the principal author of the library. ``` ## Author: John W. Eaton <jwe@octave.org> ``` Maintainer: This line should contain a single name/address as in the Author line, or an address only, or the string jwe. If there is no maintainer line, the person(s) in the Author field are presumed to be the maintainers. The example above is mildly bogus because the maintainer line is redundant. The idea behind the Author and Maintainer lines is to make possible a function to “send mail to the maintainer” without having to mine the name out by hand. Be sure to surround the network address with <...> if you include the person's full name as well as the network address. Created: This optional line gives the original creation date of the file. For historical interest only. Version: If you wish to record version numbers for the individual Octave program, put them in this line. Adapted-By: In this header line, place the name of the person who adapted the library for installation (to make it fit the style conventions, for example). Keywords: This line lists keywords. Eventually, it will be used by an apropos command to allow people will find your package when they're looking for things by topic area. To separate the keywords, you can use spaces, commas, or both.

Just about every Octave function ought to have the Author and
Keywords header comment lines. Use the others if they are
appropriate. You can also put in header lines with other header
names—they have no standard meanings, so they can't do any harm.
